# Follow‐up of patients subjected to direct and indirect pulp capping of young permanent teeth. A retrospective study

**DOI:** 10.1002/cre2.362

**Published:** 2020-12-31

**Authors:** Carmen Llena, Miriam Hernández, Maria Melo, José Luis Sanz, Leopoldo Forner

**Affiliations:** ^1^ Departament of Stomatology Universitat de València Valencia Spain

**Keywords:** direct pulp capping, indirect pulp capping, Vital pulp therapy, young permanent teeth

## Abstract

**Objective:**

A retrospective study of the success rate of direct pulp capping (DPC) and indirect pulp capping (IPC) was carried out in children between 6–14 years‐old, considering separately primary caries or caries affecting teeth with molar incisor hypomineralization (MIH).

**Material and methods:**

Data were collected in a dental public health service. Following the inclusion criteria, 232 treatments were analyzed. Success was defined by the presence of a functional tooth without clinical signs or symptoms of pulpal or periapical disease. The success rate was correlated to patient gender, the affected tooth and the indication of therapy using the chi‐squared and Fisher exact test. The success time related to treatment type was evaluated through the Mann–Whitney test.

**Results:**

The IPC and DPC success rate was 99.4%, and 84.6%, respectively (*p* = .01). Success was significantly lower when caries affected teeth with MIH than when caries affected teeth without MIH (*p* = .01). The mean survival for DPC and IPC was 14.07 ± 1.30 and 15.98 ± 0.80 months, respectively (*p* = .07).

**Conclusions:**

When caries were located in teeth that were not affected by MIH, IPC was significantly more successful than DPC, but did not differ significantly when caries were placed in teeth with MIH.

**Key points**

- Minimally invasive therapy is a successful approach for decayed young permanent teeth.
- The success of IPC was greater than the success of DPC wen caries was placed in teeth not affected by MIH.
- In teeth not affected by MIH the success of DPC or IPC did not differ significantly.

## 1 INTRODUCTION

Dental caries is a chronic multifactorial disease that shares risk factors with other chronic disorders and results in mineral loss of the hard tissues of the tooth as a consequence of the action of acids produced by bacteria of the biofilm adhered to the dental surface secondary to the metabolization of diet carbohydrates (Flick & Marchini, 2019). Caries is highly prevalent, affecting over 80% of the world population, and constitutes a public health problem in all countries. On the other hand, people are susceptible to dental caries throughout life, and the disorder is moreover cumulative (Bowen, 2016). Caries is associated with important direct and indirect economic costs, and in most countries treatment of the disease is either not covered or is only partially covered by public healthcare services (Flick & Marchini, 2019).

Carious lesions are developed as a result of subsurface demineralization of enamel, if this process is allowed to continue, the carious lesions progress, causing collapse of the enamel surface and the appearance of cavitation. The dentinal layer, cement and pulp become affected over time (Fejerskov, 2004). Development of these lesions is a dynamic process, with alternative periods of progression, arrest and regression (Nyvad et al., 2013).

Young permanent teeth (YPT) are characterized by recent eruption and incomplete apical development. Immature teeth are defined as those that have been in the mouth for less than three years, with incomplete apical development, and root walls that have not yet been fully formed (Hatami & Dreyer, 2019). Caries is one of the most frequent factor that affects these teeth, mainly first permanent molars, compromising the dental pulp. Treatment always seeks to preserve pulp vitality and secure complete root development (Camp, 2008). The maintenance of pulp vitality is essential in order to allow correct development of the tooth apex and root, and it is facilitated by the fact that YPT have a high repair capacity (Hilton, 2009).

The assessment of pulp tissue condition in YPT requires a correct clinical diagnosis, with compilation of the full medical and dental history of the patient, placing special emphasis on the pain background. A complete clinical exploration is required, including palpation, percussion and complementary tests such as radiographs. In the event of accidental pulp exposure, direct visual inspection of the pulp is indicated, since the presence of bright red pulp tissue with controllable bleeding is indicative of pulp vitality (Rosenberg et al., 2009).

The regenerative capacity of the YPT is greater than that of mature teeth (American Academy on Pediatric Dentistry Clinical Affairs Committee‐Pulp Therapy Subcommittee, American Academy on Pediatric Dentistry Council on Clinical Affairs, 2008), so conservative techniques have high probability of preserving pulp vitality and root development (Agrafoti et al., 2005). These conservative techniques are only feasible in the absence of clinical or radiological evidence of irreversible pulp disease, although some authors propose to perform conservative treatments, even in the presence of apical areas, using mineral trioxide aggregate (MTA) or other bioactive materials for pulp coating, with a success rate around 76% (Linsuwanont et al., 2017)^.^

Calcium hydroxide (CH) has been considered the “gold standard” of direct pulp capping (DPC) materials (Hilton, 2009). MTA has also been widely used as a DPC material. MTA includes a Portland cement component and shows antibacterial activity through the release of CH, which explains why it has a similar action as conventional CH (Reston & De Souza, 2009). An ideal pulp capping material should adhere to tooth structure, maintain a sufficient seal, be insoluble in tissue fluids, dimensionally stable, nonresorbable, nontoxic, noncarcinogenic, nongenotoxic, radiopaque, and exhibit biocompatibility and bioactivity. The recently developed bioactive glasses show some of these properties that make them suitable for vital pulp therapy. These materials exhibited hydroxyapatite‐like precipitation, a stable pH level, biocompatibility and bioactivity, without exhibiting cytotoxic effects (Camilleri & Pitt, 2006).

Vital pulp therapy requires isolation with a rubber dam, minimizing bacterial contamination and favoring reparative dentin formation in the lesion, clinical and radiological follow‐up, and vitality monitoring. These minimally invasive procedures in the restorative management should be the options of choice in routine dental practice, since they serve to avoid more invasive treatments, and are more biological in nature (Schwendicke & Göstemeyer, 2016).

The techniques available for the conservative management of deep carious lesions, seeking to preserve pulp vitality, comprise indirect pulp capping (IPC), direct pulp capping (DPC), and partial pulpotomy (PP) (Asgary et al., 2013).

As far as we know, few studies conducted in the context of public health programs have evaluated the middle and long term clinical outcomes of treatments due to deep carious lesion in YPT secondary to caries, using DPC or IPC, and the possible factors conditioning the success or failure of such treatments (Alqaderi et al., 2014; Gurcan & Seymen, 2019).

The aim of this retrospective study was to evaluate the success rate of DPC and IPC in children between 6–14 years‐old, considering separately primary caries or caries affecting teeth with molar incisor hypomineralization (MIH).

## 2 MATERIAL AND METHODS

A retrospective study was carried out in a primary care dental health service of the University General Hospital (Valencia, Spain). The study follows the Strobe statement for cohort studies. All experimental protocols were carried out in accordance with Helsinki guidelines and regulations. The study was approved by the Ethic Committee of the University of Valencia (H1509557670335). Permission was granted by the Primary Care Authorities to use the clinical information of the patients. When the children visited the clinic for routine controls, informed consent was obtained from their parents or caregivers in order to use the data contained in the clinical histories.

The study sample was recruited among the children between 6 to 14 years‐old, subjected to DPC and IPC in YPT in the mentioned dental service during the period between January 2014 and December 2018. Data collected were age, gender, dental group, and caries (ICDAS codes 4–6). It was noted if the caries were on a tooth affected by MIH, coded according to the Weerheijm criteria (Weerheijm et al., 2003).

The inclusion criteria were: children with at least 6 years‐old, permanent teeth exhibiting primary deep caries lesion (ICDAS codes 4–6), involving half or more of the dentine (detected by radiographic examination), absence of spontaneous pain, tenderness on percussion or palpation, positive response to cold testing (Endo‐Ice, Hygenic Corp, Akron, OH), absence of radiolucent periapical lesions, minimal pulp exposure (if any) with control of bleeding by applying pressure with a cotton pellet for 5 min. The size of the exposure was not specifically measured, it was interpreted to be minimal when clinically it did not present a size larger than the diameter of a 1.5 mm burr. This was a criteria for including the teeth with those size of exposures in the follow‐up evaluation.

Exclusion criteria were: patients with general disease and those who suffered pulp exposure of over 1.5 mm (still meeting the initial criteria), or in which bleeding upon exposure could not be controlled within 5 min.

The diagnosis, treatment procedures and follow‐up, were carried out by a single operator, this allowed that all treatments were performed with the same clinical protocol. The data from the clinical records were gathered by a different researcher than the one who performed the treatments and follow‐up. Therefore since the data from the previously performed diagnosis, treatment and follow‐up were extracted from the clinical records the study design is retrospective.

After anesthesia and isolation with a rubber dam, the cavity was opened with a diamond bur. The walls of the cavity were cleaned using rotary and manual instruments until hard dentin was reached. Partial removal of the soft carious tissue from the cavity floor was carried out with excavators until the lathery dentine was reached (Schwendicke & Göstemeyer, 2016). In the event of 1–1.5 mm pulp exposure during this process, the cavity was cleaned with 0.2% chlorhexidine and bleeding control was controlled with a cotton ball during 5 min. If bleeding control proved successful, pure CH was placed, (Calcipulpe, Septodont, Mataró, Spain) followed by a photocured glass ionomer (Vitrebond 3 M, Saint Paul, MN) and a composite resin (Ceram X Universa Dentsply Sirona, Erlangen, Germany) or silver amalgam restoration (Permite, SDI, Victoria, Australia) (DPC). If bleeding control was not achieved, or in the event of a pulp exposure greater than 1.5 mm, a PP was performed or conventional canal treatment was provided, and the affected tooth was excluded from the study analysis.

In the absence of pulp exposure, the cavity was cleaned with 0.2% chlorhexidine during one minute, glass ionomer (Vitrebond 3 M, Saint Paul, MN) was placed, and a coronal composite resin or silver amalgam restoration was performed (IPC).

Follow‐up visit were planed every 6 months. The clinical success criteria after a minimum 6‐months follow‐up period were the presence of a functional tooth without clinical signs or symptoms of pulp or periradicular disease (no spontaneous pain or hypersensitivity to cool, heat or sweets, no pain in response to palpation and/or percussion, no sinus tract, soft tissue swelling or mobility). The presence of any of these signs or symptoms was considered indicative of failure (Brodén et al., 2019).

### 2.1 Statistical analysis

The associations between the pretreatment variables and treatment success were explored using the chi‐squared test and Fisher exact test. Survival time was related with treatment type by Mann–Whitney test. Statistical significance was considered for *p* < .05. The SPSS version 24.0 statistical package (IBM Corp., Armonk, NY) was used.

## 3 RESULTS

**FIGURE 1:**
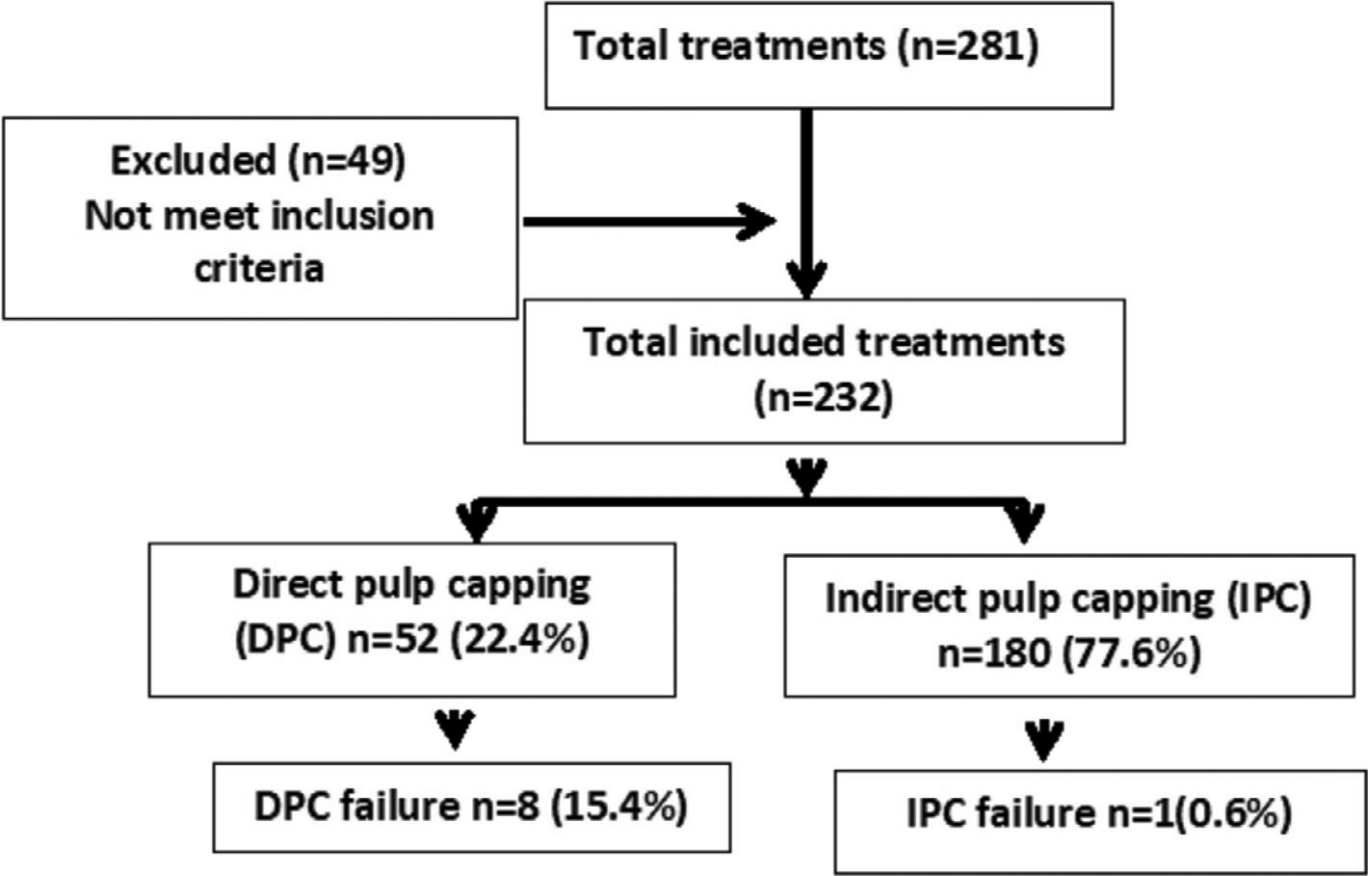
Flow diagram representing the follow‐up of patients which met the inclusion criteria, reasons for exclusion, and the total number of evaluated teeth

Data from 281 DPC and IPC treatments were collected, but we included those cases with a minimum follow‐up of 6 months (232 treatments in 170 patients), with a range from 6 to 36 months. A flow diagram representing the follow‐up of patients which met the inclusion criteria, reasons for exclusion, and the total number of evaluated teeth, is presented in Figure 1. The mean age was 9.8 ± 2.2 years, and the mean follow‐up was 15 ± 9.2 months. The gender distribution was balanced (50.3% males and 49.7% females). Most of the treated teeth (94.8%) were first molars (220 cases), while 2.6% were premolars (6 cases) and 2.6% incisors (6 cases). With regard to the type of treatment, 77.6% corresponded to IPC (180 cases) and 22.4% to DPC (52 cases). In 53 cases (22.9%) caries was located on a tooth with MIH. Percentage and type of failure are described in Table 1.

**TABLE 1:** Percentage of cases showing signs or symptoms of failure

	Total	DPC	IPC
Spontaneous pain	5 (2.7%)	5 (11.8%)	—
Swelling	1 (0.5%)	1 (2.3%)	
Cool sensitivity	4 (2.2%)	3 (7%)	1 (0.7%)
Presence of sinus tract	3 (1.6%)	3 (7%)	—
Radiographic periapical lesions	1 (0.5%)	1 (2.4%)	—

Abbreviations: DPC, direct pulp capping; IPC, indirect pulp capping.

**FIGURE 2:**
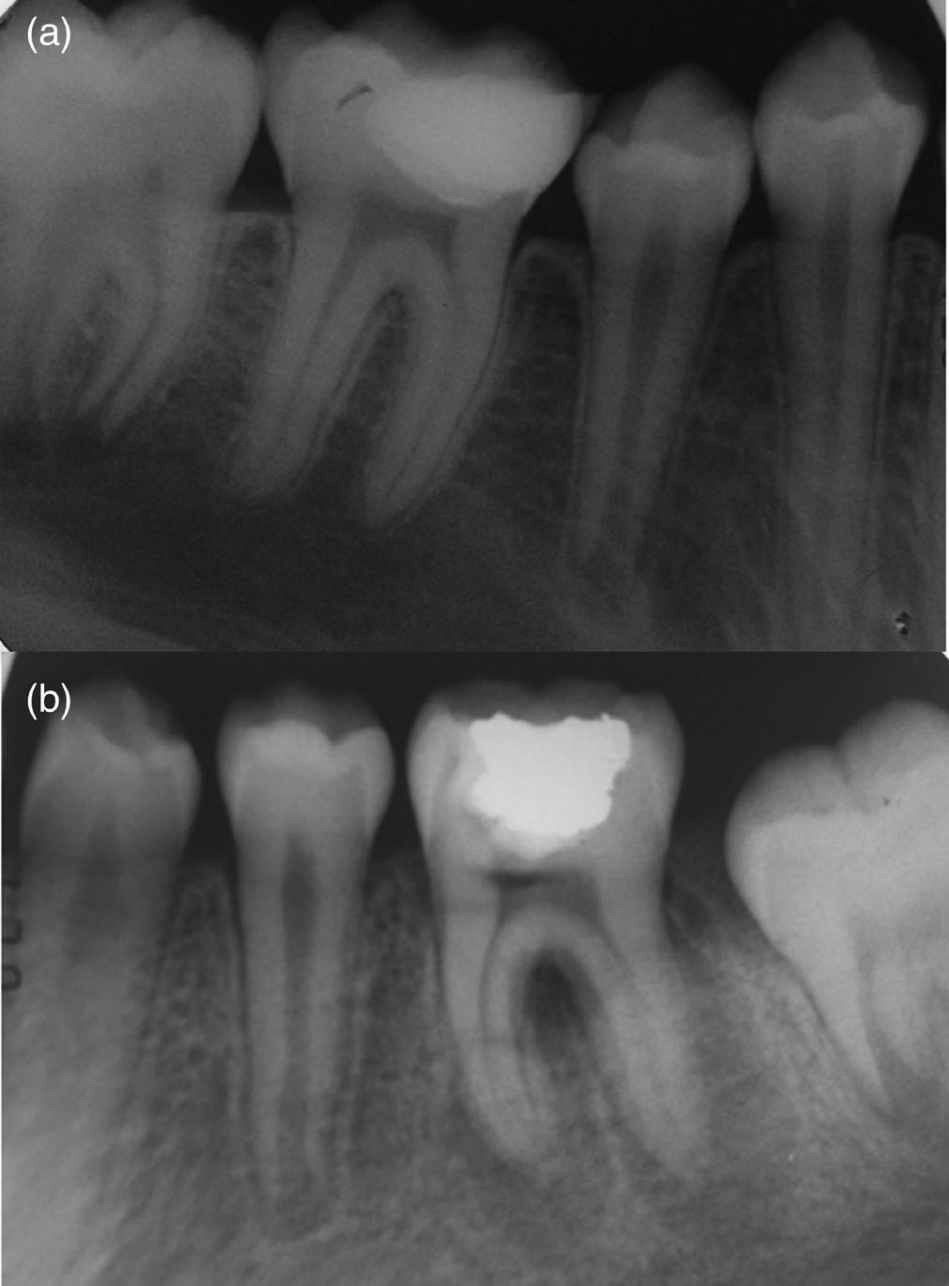
(a) DPC with X‐ray satisfactory status. no apical lesion is observed (b) DPC with X‐ray failure status. Success after 2‐years follow‐up. no apical lesion is observed (a). Failure after 12‐months follow‐up. A periapical radiolucency is observed in mesial and distal roots (b)

Figure 2a shows a DPC, whose clinical and radiographic status was satisfactory after 2 years of follow‐up. Figure 2b shows the image of a case of failure of a DPC after 12‐months follow‐up.

The mean age of children with caries that affects a tooth without MIH was 10.3 ± 2.2 years‐old, and in cases with caries associated with MIH was 8.3 ± 1.6 years‐old (*p* < .01). The mean ages of children treated with DPC and IPC were 9.6 ± 1.2 years and 9.8 ± 0.9 years, respectively, without significant differences.

The type of treatment and the indication of therapy were significantly related (*p* = .02). Percentage of DPC was higher than IPC when caries affected teeth with MIH (Table 2). A significant association was also found between the treated teeth and the type of treatment (Table 3) (*p* < .01). molars were the most commonly treated teeth for both DPC and IPC.

**TABLE 2:** Type of treatment related to the cause (*p* = .02)

	Caries	MIH
DPC	36 (20.1%)	16 (26%)
IPC	143 (79.9%)	37 (74%)

Abbreviations: DPC, direct pulp capping; IPC, indirect pulp capping; MIH, molar incisor hypomineralization.

**TABLE 3:** Type of treatment related to the teeth (*p* < .01)

	Incisor	Premolar	Molar
DPC	6 (11.5%)	1 (1.9%)	45 (86.5%)
IPC	0	5 (2.8%)	175 (97.2%)

Abbreviations: DPC, direct pulp capping; IPC, indirect pulp capping.

The percentage of failure rate was 15.4% and 0.6% for DPC and IPC, respectively. The mean survival for teeth treated DPC was 14.07 ± 1.30 months, versus 15.98 ± 0.80 months in the case of IPC. The median was 12 months for both treatments. The difference failed to reach statistical significance (*p* = .07).

The percentage of success for IPC was 99.4% and for DPC 84.6% (*p* < .01). The percentage of success in the case of caries was of 97.2%, and in MIH was 94% (*p* = .27).

For each type of treatment, we evaluated success percentage according to the indication of therapy. As can be seen in Table 4, in those cases where the indication was caries, IPC showed to be significantly more successful than DPC (*p* < .01). However, when the indication of therapy was caries affecting a tooth with MIH, no significant differences were observed between the two types of treatment (*p* = .568).

**TABLE 4:** Success by type of treatment and cause (*p* < .01)

	Caries	MIH
DPC	31 (86.1%)	13 (81.3%)
IPC	143 (100%)	36 (97.3%)

Abbreviations: DPC, direct pulp capping; IPC, indirect pulp capping; MIH, molar incisor hypomineeralization.

The number of teeth evaluated in each period were 86 (6 months), 61(7–12 months), 57 (13–24 months), 28 (>24 months). Only the latter follow‐up visits were considered for the evaluation.

**FIGURE 3:**
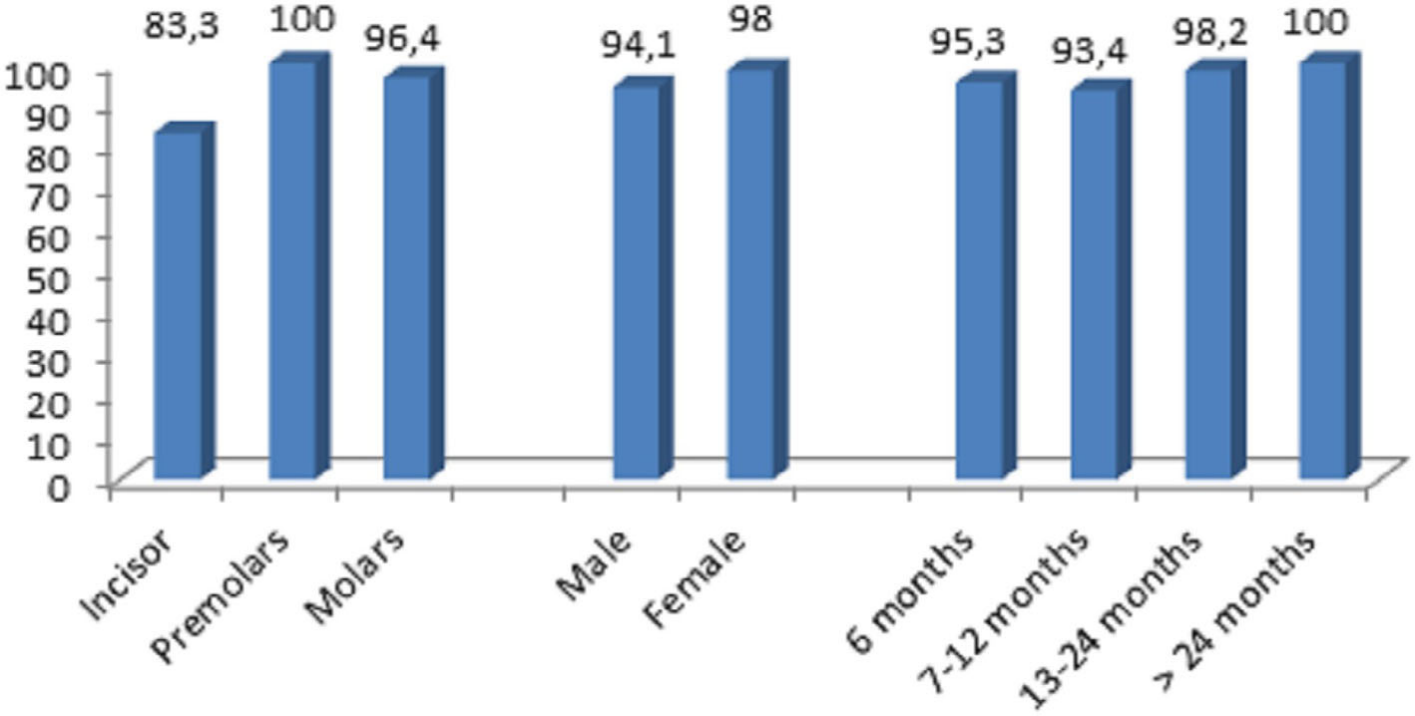
Success rate percentage according gender, type of tooth, and follow‐up period

No statistically significant association was found between treatment success and gender, type of tooth, or follow‐up period *p* > .05 (Figure 3).

## 4 DISCUSSION

The present study evaluates treatment success in patients with deep carious lesion using criteria for minimally invasive management or selective caries removal. DPC was used in the case of accidental pulp exposure during infected tissue removal. The classical technique was used, with the application of CH, followed by glass ionomer and definitive restoration in a single step, while IPC was used in the absence of pulp exposure, applying glass ionomer and a definitive restoration, likewise, in a single step. These procedures were carried out in the context of a public community care program.

Despite the evidence supporting the advantages of selective caries treatment in a single step or in two steps versus complete caries removal, the latter procedure is still very often used by dental professionals. A recent systematic review indicates that 53% of all dentists decide a complete removal of carious tissues in patients with deep carious lesions (Schwendicke & Göstemeyer, 2016). The lack of clear protocols and issues referred to training is, possibly, a determinant factor leading many dental professionals to choose a less conservative management approach.

In this clinical study all treatments were performed in one step. We chose single step treatment due to the time saving and the lesser discomfort for the patient. Moreover, single‐step therapy avoids the risk of patient failure to return for the second treatment session and lessens the risk of pulp exposure. On the other hand, the results of treatment, in terms of maintaining pulp vitality, carried out in a single step versus two‐step show more favorable results when the treatment is performed in a single step (Brodén et al., 2019; Maltz et al., 2012).

The present study included all teeth requiring restorative treatment in the period between January 2014 and December 2018, with dentinal involvement to the internal third caused by caries. We decided to differentiate carious lesions affecting teeth with MIH because patients with severe forms of this type of disorder show a higher prevalence of caries, extensive and deep lesions, and a greater need for treatment (Kosma et al., 2016). As far as we know, no studies have been evaluated success differences in caries that affected teeth with MIH or caries in teeth not affected by MIH.

All the diagnoses and treatments were made by a single operator and under isolation using a rubber dam, as advised in order to minimize the risk of contamination (American Academy on Pediatric Dentistry Clinical Affairs Committee‐Pulp Therapy Subcommittee, American Academy on Pediatric Dentistry Council on Clinical Affairs, 2008).

The studies found in the literature differ in terms of sample size and the follow‐up duration. The available series typically report between 50 and 300 treatments, with a 6 months to 10 years follow‐up (Schwendicke et al., 2013). In our series, follow‐up time ranged between 6 and 36 months, and 232 teeth from the 281 treatments performed during the study period were included in the analysis.

The criteria used to assess success were the absence of clinical signs and symptoms of pulp or periapical disease. Although some studies included radiographic criteria, such as apical sealing or the absence of radiolucencies (Schwendicke et al., 2013), we only considered clinical data, since the absence of clinical signs and symptoms in young teeth without previous apical disease was taken to be indicative of a good outcome. Moreover, pediatric patient exposure to X‐rays should only occur if fully justified. Radiological evaluations were made in our series when required by the clinical signs and symptoms (American Academy of Pediatric Dentistry ad hoc Committee on Pedodontic Guideline on Prescribing Dental Radiographs for Infants, Children, 2012).

Pulp tests were not included for evaluation in the follow‐up, because it is not systemically included in the clinical protocol of primary care dental health service. It was assumed that a patient that did not refer clinical symptoms of hypersensitivity, nor presented spontaneous pain or pain to palpation or percussion has a favorable evolution. However, this turns out to be a limitation in this investigation.

A systematic review conducted by Schwendicke et al. (2016), concluded that the use of different materials for pulp capping is supported by only a limited number of controlled clinical trials. Most materials did not show superiority compared with CH. While MTA might be a valuable alternative to CH for DPC, no firm evidence was reached to strongly support any recommendations in permanent teeth. For this reason, from the point of view of public health, considering the cost benefit, CH may be a therapeutic alternative in DPC.

Our study considered the clinical efficacy of CH followed by glass ionomer and restoration in the case of DPC and those of glass ionomer and restoration in the case of IPC. CH was used in the former case because it has been the gold standard for pulp preservation treatment for many years. Brizuela et al. (2017) compared different materials in DPC (CH, Biodentine and mineral trioxide aggregate (MTA)), and they found no significant differences among the three materials. Their percentage success rate after one year of follow‐up was 86.5%, which is very similar to our own rate of 86.7% at the end of the follow‐up. Taking into account that they included teeth with opened and closed apexes like us.

Katge and Patil (2017), reported a 100% success in permanent molars with open apex using Biodentine or MTA. In the present study, not all the teeth had an open apex, what could have influenced our lower success, in addition to the difference in the material. Other studies involving young and adult patients, reported success rate of DPC ranges between 62%–80% (Hilton et al., 2013). Such variability may be attributed to the different pulp tissue response in YPT compared with mature teeth (Hilton, 2009).

Some authors consider that exposure should not exceed 1 mm and that the outcomes are more favorable in young teeth (Schwendicke et al., 2016). In our study pulp exposure was approximately 1 mm, and we only included those cases in which correct bleeding control was achieved after exposure.

With regard to dentin‐bridge forming capacity, a histological study published by Sawicki et al. (2008) in premolars removed due to orthodontic reasons observed no significant differences between CH and MTA. Usually, the increase in extracellular calcium resulting from the use of calcium‐mimetics and calcium‐based pulp capping materials promotes osteogenic and odontogenic differentiation and mineralization.

In the case of IPC treatments with glass ionomer, the percentage success rate was 99.4%, which is similar to the data reported by Gurcan and Seymen (2019) who compared CH, glass ionomer and MTA. These authors found a similar success percentage for all three materials after 6 months, with comparable dentin‐bridge formation as assessed by cone‐beam computed tomography (CBCT).

In a study of patients with different ages, Maltz et al. (2012) found a 91% of success rate after partial caries removal and IPC. They also compared this technique with the stepwise or gradual excavation procedure and concluded that the final outcome, in terms of the preservation of pulp vitality, was superior with partial caries removal. In clinical practice, both the stepwise technique and partial caries removal resulted in increased preservation of pulp vitality when compared with total caries removal procedures (Schwendicke et al., 2013).

Hashem et al. (2015) compared Biodentine with a glass ionomer in IPC treatments of molars and premolars with deep carious lesions and recorded a success rate of 83.3% with both materials. Differences with our results could be explained by the fact that the aforementioned researchers included patients of different ages, not only children.

Our research shows how in patients with caries that affect a tooth with MIH the percentage of DPC was significantly greater than that of IPC. Considering the two types of treatment procedures, the percentage of success rates were greater when teeth were not affected by MIH. Dentin under enamel has a greater amount of interglobular dentin with a 5% reduction of mineralization. The term interglobular dentin designates the organic matrix that remains nonmineralized because the mineralization globules do not fuse. This is most frequently observed at the level of secondary dentin immediately below the dentin of the mantle, where the mineralization pattern is more likely to be globular rather than apposition. This greater presence of interglobular dentine could be the cause why caries lesions in teeth affected by MIH were more extensive than in teeth not affected by MIH, and therefore needing more DPC treatments (Heijs et al., 2007). The least success in decayed teeth affected by MIH could be explained by the inflammatory changes in the pulp of these teeth. Gatón‐Hernández et al. (2020) evaluated success of selective removal of carious tissue, in teeth affected by MIH after 24 months follow‐up, finding a success rate of 96.8%, very closely to showed in the present study.

None of the factors evaluated in the present study were significantly associated with the success of DPC or IPC, only the presence of MIH in a tooth that was later affected by caries showed a tendency to obtain worse results with both types of treatment. Future studies should include larger samples, longer follow‐up periods, study of pulpal inflammation status, and inclusion of teeth with both open and closed apexes to evaluate the long term success of treatment.

Despite the intrinsic limitations of clinical studies, it may be concluded that IPC showed higher percentage of success rate than DPC. Success was greater in teeth not affected by MIH. When caries were located in teeth that were not affected by MIH, IPC was significantly more successful than DPC, but did not differ significantly when caries were placed in teeth with MIH.

## CONFLICT OF INTEREST

The authors declare that they have no conflict of interest.

## AUTHOR CONTRIBUTIONS

Carmen Llena and Leopoldo Forner conceptualization, design and data analysis. Miriam Hernández and Maria Melo collection and data processing. José Luis Sanz writing the text.

## Data Availability

Author elects to not share data.
